# Program development and initial experiences with the online class “Erste Hilfe Kasten – Allgemeinmedizin”

**DOI:** 10.3205/zma001741

**Published:** 2025-04-15

**Authors:** Sabine Seiler, Frederik Schelter, Eberhard Nöfer, Marco Roos

**Affiliations:** 1 Universität Augsburg, Medizinische Fakultät, Allgemeinmedizin, Augsburg, Germany; 2 Hochschule für angewandte Wissenschaften Coburg, Integrative Gesundheitsförderung, Coburg, Germany

**Keywords:** general medicine, postgraduate training, e-learning, management

## Abstract

**Objective:**

This project aims to provide doctors in postgraduate training (ÄiW) in general medicine with business knowledge of practice management through a modular training program within an e-learning environment, thereby reducing important barriers to establishing a medical practice.

**Methodology:**

To develop the concept, a needs analysis (interviews with medical specialists, ÄiW, and medical students) was combined with teaching and informational materials from the Association of Statutory Health Insurance Physicians.

**Results:**

The developed e-learning concept consists of twelve self-contained modules that include various formats (teaching letters, videos, audio slide presentations, and checklists) for knowledge transfer and reflection. By May 2024, 72 participants had started using the class. The needs initially stated by the participants largely corresponded with the content offered. So far, the possibility of feedback to adapt the content or questions about the content have not been used.

**Conclusion:**

The modular e-learning class presented here covers business management topics in general practice and can help reduce this important barrier.

## Zusammenfassung

**Zielsetzung:**

Ziel dieses Projekts ist es, Ärztinnen und Ärzten in Weiterbildung (ÄiW) in der Allgemeinmedizin durch ein modulares Ausbildungsprogramm in einer E-Learning-Umgebung betriebswirtschaftliche Kenntnisse in der Praxisführung zu vermitteln und dadurch eine wichtige Barriere zur hausärztlichen Niederlassung zu reduzieren.

**Methodik:**

Zur Konzeptentwicklung wurde eine Bedarfsanalyse (Interviews mit Fachärztinnen und Fachärzten, AiW und Medizinstudierenden) mit Lehr- und Informationsmaterialien der ärztlichen Selbstverwaltung kombiniert.

**Ergebnisse:**

Das entwickelte E-Learning-Konzept besteht aus zwölf in sich geschlossenen Modulen, die verschiedene Formate (Lehrbriefe, Videos, vertonte Folienpräsentationen und Checklisten) zur Wissensvermittlung und Reflexion beinhalteten. Bis Mai 2024 haben insgesamt 72 Teilnehmende das Angebot zu nutzen begonnen. Die von den Teilnehmenden initial genannten Bedarfe decken sich in hohem Maße mit den angebotenen Inhalten. Bisher wurde die Möglichkeit eines Feedbacks zur Anpassung der Inhalte oder Fragen zum Inhalt nicht genutzt.

**Schlussfolgerung:**

Mit dem hier vorgestellten modularen E-Learning-Angebot liegt ein Konzept vor, das betriebswirtschaftliche Themen der hausärztlichen Niederlassung abdeckt und einen Beitrag leisten kann, diese wichtige Barriere zu reduzieren.

## Introduction

Primary care close to home is at stake. Demographic developments and the shortage of skilled workers indicate a gap in demand of up to 3,000 general practitioners (GP) in the next ten years [1]. Working as a GP seems appealing to students [2]. However, running a practice as newly qualified specialists is made more difficult by their fears of economic responsibility, possible claims for recourse, a higher workload, and a lack of flexibility in work arrangements [3], [4], [5], [6], [7], [8]. A training program for doctors in postgraduate training (ÄiW) in primary care that introduces them to the basic principles of business administration is not regularly available.

## Project description

The project presented here aims to impart business management knowledge in setting up and managing a practice in primary care through self-directed e-learning and thus reduce one of the most important barriers.

### Concept planning and development

**Figure 1:**
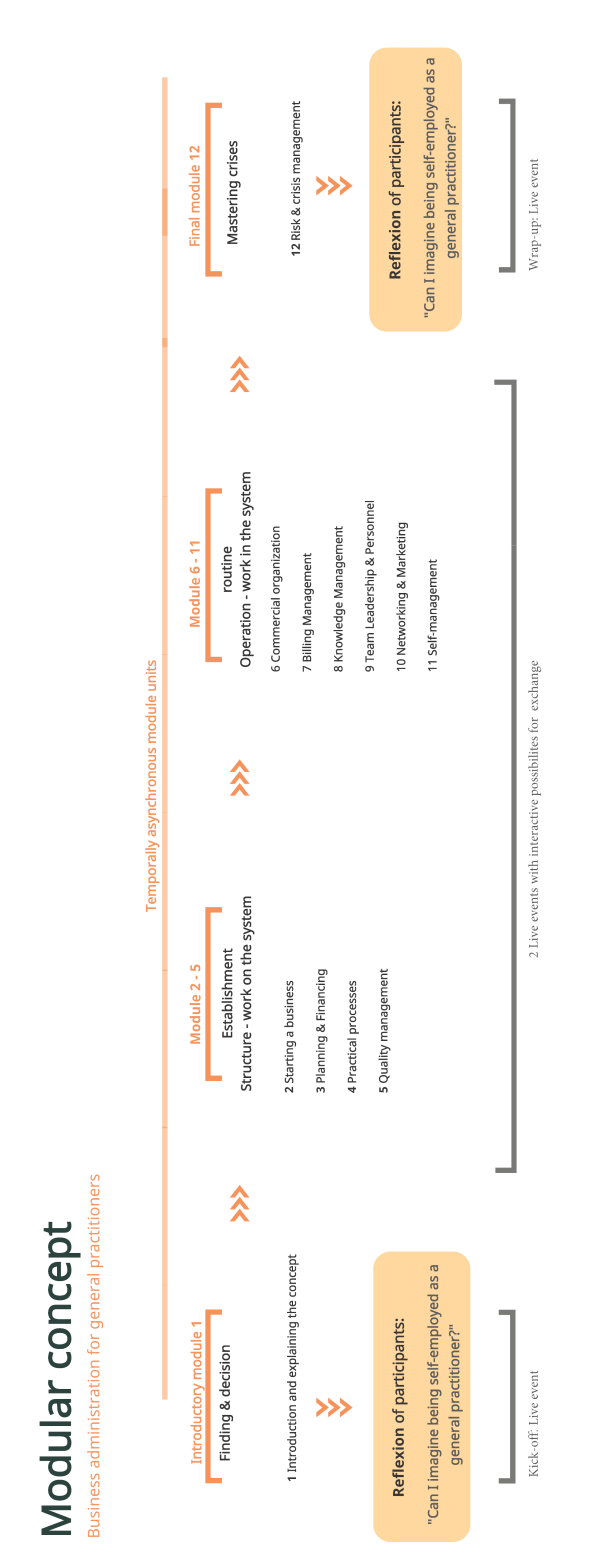
Modular concept at a glance

**Figure 2:**
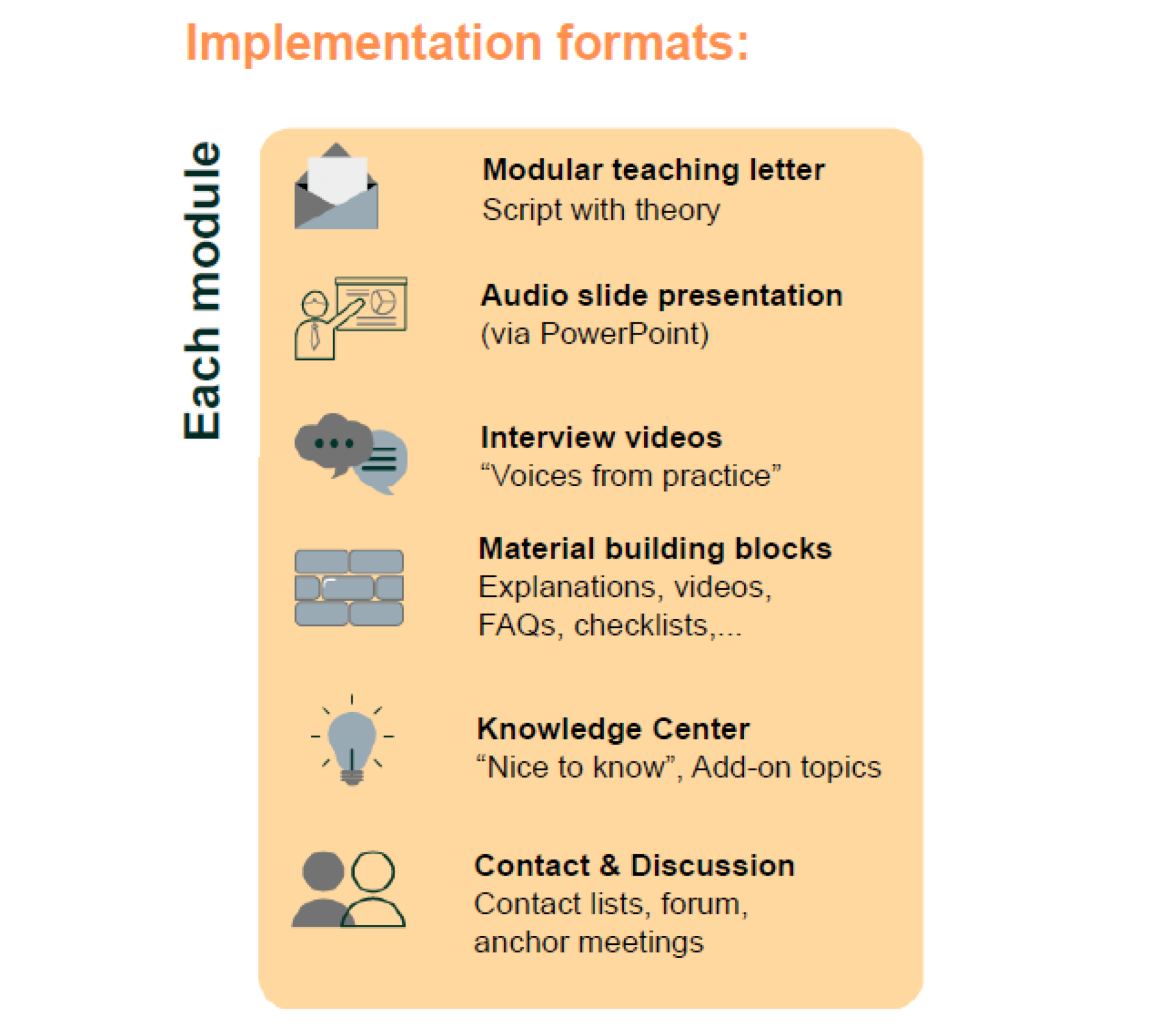
Formats of the modular concept

According to the core cycle for curriculum development, a needs analysis was first carried out [8]. For this purpose, interviews were conducted with GPs (ex post view), ÄiW and medical students (ex ante view). The results were compared and supplemented with content from teaching and information materials from the Association of Statutory Health Insurance Physicians. The resulting concept consists of twelve modules (see figure 1 (Fig. 1)). Didactically, on the one hand, a learning path was created that includes the activation of prior knowledge (video sequences with expert opinions from practice or short questionnaires for self-assessment), provides impulses for building up knowledge (such as teaching letters, audio slide presentations), and encourages reflection/application (summaries or checklists for transfer to your situation) (see figure 2 (Fig. 2)). On the other hand, a learning environment was also developed that offers different options for self-study depending on the learning style preference. The Bavarian Competence Center for Specialty Training in General Practice (KWAB) makes the new class available. 

The module concept is uploaded to an online learning platform (initially ILIAS^©^, now Moodle). The goal is to complete the program in 12 weeks. The modules are self-contained and can also be worked through separately. The learning environment should give the learners maximum freedom in the individual processing of the module units.

### First experiences with the online class

The online class for ÄiW has been accessible and free of charge since December 2023 [https://iam-augsburg.de]. By the end of May 2024, 72 participants had started the program. Of these, 72 (55% female, 45% male) completed the initial survey. Over two-thirds of the participants undergo further general medicine training (see table 1 (Tab. 1)).

In the beginning, participants have the opportunity to express their needs regarding practice management in free text answers. A total of 101 needs have been specified thus far. With around a quarter of the responses, the participants see the most significant gaps in practice management in business management topics.

After that in response, at least 10% of the mentions are related to billing, human resources management, and legal topics (see table 2 (Tab. 2)).

## First conclusion

The e-learning class presented here offers those in postgraduate training who want to become a GP the opportunity to gain an overview of all relevant topics in practice management with 12 modules. In addition, we observed that those from other disciplines also use the public class to establish a registered medical practice. This addresses a barrier to establishing a practice that has been mentioned several times in the literature in the German context [3], [4], [5], [6], [7], [8]. An advantage of the concept presented here could be the possibility of editing regardless of location and time. 

The participants’ initially mentioned needs for practice management correspond to a large extent with the content of the modules. Of course, it must be critically examined whether participation is used in the sense of a selection bias by those who perceive a high personal deficit or feel insecure about these topics. 

Possible challenges could be responded to by involving the target group and those working in statutory healthcare at an early stage in the needs analysis of the conception phase. Additionally, consistency within existing literature ensured that potential issues were addressed effectively.

As a limitation, it must be noted that the e-learning class is an opportunity for self-study and, therefore, does not include any practical application or discussion with colleagues. However, the participants have not yet requested this option, although this is possible in the modules. It also seems plausible that the participants use the class to gain an overview of the topics. This means that participants can specifically take advantage of other classes, such as the seminar series of the “Werkzeugkasten Niederlassung für Hausärztinnen und Hausärzte” of the General Practitioners Association or the offers of the Association of Statutory Health Insurance Physicians.

## Take home message

The modular e-learning concept presented here can help reduce the important barrier of business management topics in general practice by covering them in the modules of this class. 

## Notes

### Author’s ORCID

Marco Roos: [0000-0003-1596-5908]

### Funding

The study was financially supported by the Bavarian State Ministry of Health, Care and Prevention (StMGP). (Bescheid Nummer: G31c-G8060-2018/40-13).

## Acknowledgements

We would like to thank everyone who contributed to the content of this offer. Our special thanks go to Dr. Beate Reinhardt and Prof. Astrid Nöfer, through whose initiative and idea this project came about.

## Competing interests

The authors declare that they have no competing interests. 

## Figures and Tables

**Table 1:**
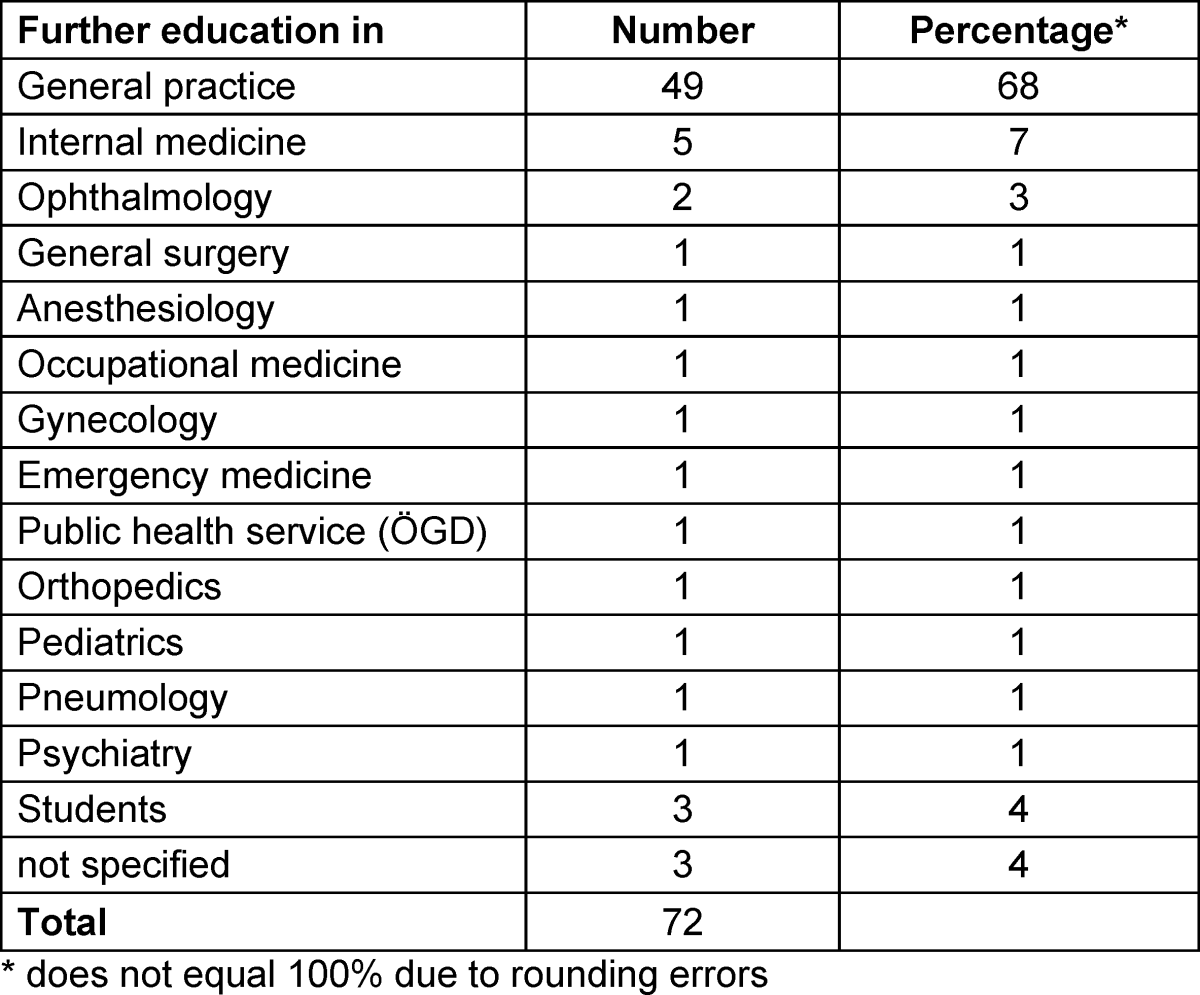
Information about the further training of the participants

**Table 2:**
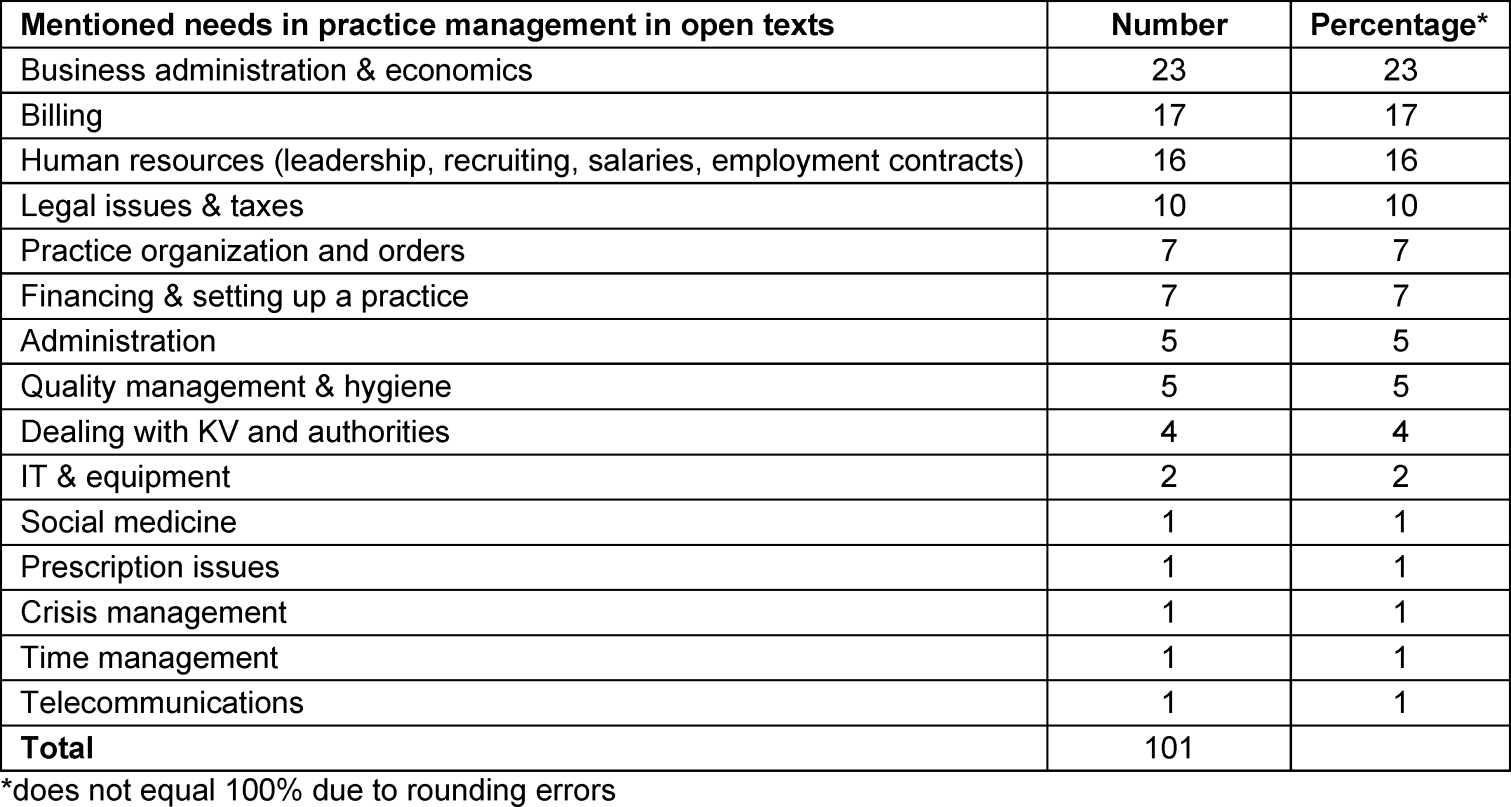
Mentioned needs in practice management in open texts
